# Promoting the Cation Utilization in Energy‐Dense Sodium Metal Battery Prototypes: Strategies, Analysis, and Prospects

**DOI:** 10.1002/smsc.202300108

**Published:** 2023-11-27

**Authors:** Ting Liu, Zihan Yang, Yinwen Tang, Jintao Liu, Yue Ma

**Affiliations:** ^1^ Training Center for Engineering Practices Northwestern Polytechnical University Xi'an 710072 P. R. China; ^2^ Queen Mary University of London Engineering School Northwestern Polytechnical University Xi'an 710072 P. R. China; ^3^ State Key Laboratory of Solidification Processing, Centre for Nano Energy Materials School of Materials Science and Engineering Northwestern Polytechnical University Xi'an 710072 P. R. China

**Keywords:** cation utilization degree, dendrite formation, energy-dense model, operando characterization, sodium metal battery

## Abstract

Sodium metal batteries (SMBs) have garnered significant attention among the most promising energy storage devices due to their high theoretical energy densities and availability of charge carrier sources. However, the large volume expansion of the hostless anode and Na dendrite protrusion destabilize the solid–electrolyte interphase (SEI), meanwhile the irreversible depletion of Na^+^ ions would compromise the coulombic efficiency and lead to the unsatisfactory cation utilization during the repetitive cycling. Hitherto, a series of optimization strategies are proposed to promote SMB cation utilization, including homogenizing the cation influx toward the metallic substrate, regulating the composition of the SEI layer, and suppressing the volume propagation of metallic deposition. Most of these methods, however, are still based on empirical attempts and lack the systematic study to elucidate the interplay between the structural evolution of the electrodes and the cation utilization degree on the device level. Therefore, this review aims to consolidate the understanding of critical factors that promote the cycling efficiency and their correlations through the performance assessment on the device level. By leveraging operando characterization techniques, the future studies seek to emphasize the pivotal characteristics at multiple scales that contribute to the enhanced cation utilization degree.

## Graphical abstract

This review has provided a comprehensive analysis of strategies to promote cation utilization in energy‐dense sodium metal batteries. By identifying various influencing factors associated with the sodium metallic deposition process, particularly the root origins of nucleation and deposits propagation and their correlations, key features on the electrode level that contribute to the enhanced cation utilization are highlighted.

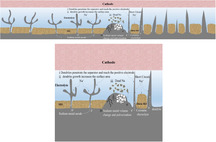

## 1 Introduction

Lithium battery technologies have dominated the energy storage market in consumer electronics, electric vehicles, and grid‐scale storage for decades.^[^ ^1^, ^2^, ^3^, ^4^ ^]^ However, the increasing demands for transportation electrification and renewable power system integration raise concerns about the scarcity of lithium resources.^[^ ^5^, ^6^ ^]^ In light of these considerations, researchers have been actively investigating alternative power solutions that afford higher energy density and environmental adaptability an alternative to the prevailing Li‐ion batteries.^[^ ^7^, ^8^ ^]^ Sodium metal batteries (SMBs) utilize low‐cost, readily available sodium cations as the carrier source, thus providing a promising energy storage format.^[^ ^9^, ^10^ ^]^ Additionally, metallic sodium exhibits a higher theoretical capacity of 1,166 mAh g^−1^ (as compared to hard carbon anodes in traditional sodium‐ion batteries) and lower electrochemical potential (−2.714 V vs standard hydrogen electrode).^[^ ^11^ ^]^ Despite of the enhanced theoretical energy density of the SMB as compared to the sodium‐ion batteries, the practical application of SMBs faces formidable challenges due to cycling inefficiencies and safety concerns.^[^ ^12^, ^13^ ^]^ These issues primarily derive from the Na^+^ reservoir depletion, which could be attributed to the sodium dendrite formation, electrolyte decomposition reactions at the electrode interface, as well as the accumulation of the dead sodium deposits. To compensate the irreversible cation loss, excessive dosage of Na^+^ sources was often employed. However, the preconfigured excess sodium source would significantly reduce the actual gravimetric/volumetric energy densities of the SMB model, meanwhile, increase the safety risks. In this regard, optimization of Na^+^ utilization is the key to solve the problem of cycling inefficiencies and secure the reliable operation of SMBs.

**Figure 1:**
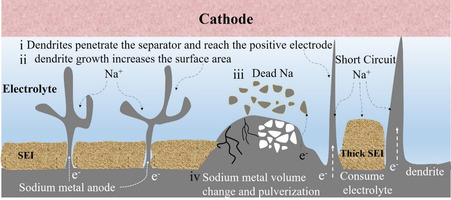
The mechanism of sodium ions depletion in SMBs.

The practical implementation of SMBs has long faced challenges due to their rapid capacity degradation stemming from sodium ions depletion. After decades of research, it came to light that the mechanism of sodium ions depletion of SMBs is attributed to the following critical issues (**Figure** **1**). The working mechanism of the SMB involves the extraction of Na^+^ ions from the cathode during the charging process. These Na^+^ ions then migrate within the bulk electrolyte along the Na^+^ concentration gradient and plate onto the anode substrate. However, the inhomogeneous deposition process can lead to sodium dendrite protrusion, which poses detrimental effects on the cyclability and operation safety of SMB models. Specifically, the accumulation of Na dendritic deposits would cause several issues:^[^ ^14^, ^15^, ^16^, ^17^, ^18^, ^19^, ^20^ ^]^ 1) Dendrites can penetrate the separator and reach the positive electrode, resulting in the short circuit scenarios, and thermal runaway, electrolyte combustion, and even battery explosion; 2) Additionally, dendrite growth increases the surface area of the negative electrode, promoting parasitic reactions between the electrode and the electrolytes. These side reactions irreversibly consume active Na^+^ ions and electrolyte, reducing the overall coulombic efficiency of the battery; 3) Furthermore, Na dendrites form a porous and uneven structure with significant amounts of inactive or “dead” sodium. This process leads to increased polarization and internal resistance; 4) The large volume expansion/contraction of metallic deposits induces the structural collapse of the anode. This continuous variation of the mechanical strength results in the electrode rupture and SEI reconstruction during the repetitive cycling, leading to deteriorated coulombic efficiency and reduced cycle life. To this end, the practical deployment of energy‐dense SMB prototypes faces formidable issues that impede the efficient utilization of Na cations within the battery system. Crucial for the coherent high energy density, long cycle life, and reliable operation of the SMB is the in‐depth understanding of the fundamental rationales as well as the deliberate regulation of sodium nucleation and propagation behaviors.

Pioneer studies involving electrode material design, electrolyte optimization, interface engineering, and battery architecture modifications have been attempted to enhance the cation utilization in energy‐dense SMB prototypes. However, most analyses have been based on indirect electrochemical analysis or isolated evaluation of the cell component. To this end, the underlying mechanisms remain obscure, resulting in merely the empirical approaches. To develop more targeted and efficient approaches, further investigation into the underlying origins of capacity fading is crucial. Advanced analysis techniques such as in situ and operando characterization methods are urgently required to provide insights into the real‐time phase/morphological evolution of the cathode, anode and multiscale interphases as well as their interplay; meanwhile, cation migration behaviors should be analyzed at the multiple scales, especially ranging from the solid‐state cathode structure, cathode electrolyte interface (CEI), in the bulk electrolyte and across the separator, SEI, finally toward the metallic deposition substrate.

Herein, this review aims to provide a comprehensive analysis of fundamental rationales, mitigation techniques, and prospects for promoting cation utilization in energy‐dense SMB prototypes. To elucidate the root origin of the dendrite formation, several nucleation mechanisms were systematically described and commented. Correspondingly, the recent modification strategies were reviewed from several aspects. First, pioneer electrolyte modifications were examined, focusing on the correlated roles of the sodium salts, types of solvents, and sacrificial additives in the liquid electrolyte. These factors significantly influence the formation process and dynamic stability of the SEI layer. Additionally, the review discussed how solid‐state electrolytes (inorganic solid electrolytes (ISEs), polymer solid electrolytes (PSEs), and composite solid electrolytes (CSEs)) with excellent mechanical properties can effectively inhibit the dendrite growth. Interfacial engineering strategies were further explored subsequently. By implementing the chemical or physical treatments, the mechanical stable SEI protection layer can be purposely constructed on the surface of the Na foil. These approaches help to prevent excessive electrolyte consumption while inhibiting the dendrite growth and volume propagation of the deposits. Thirdly, the review discussed the design ingenuity of electrode architectures. These approaches incorporate multiple complementary effects to regulate the volume changes of metal deposits and ion influx toward the Na nucleation sites. Besides, the intrinsic cell prototyping, the practical cycle behavior of SMB is significantly influenced by critical factors such as temperature, stacked pressure, current density as well as the deposition amount.^[^ ^21^ ^]^ This review provides insights into the feasible mitigation methods of cation depletion on the system level. It summarizes the various influencing factors that promote the Na^+^ utilization and their correlations based on performance evaluations of different prototypes. State‐of‐the‐art operando techniques are proposed to provide a comprehensive understanding of the key features that contribute to the cycling reversibility of the SMB. These findings offer guidance for future research endeavors that focus on emerging technologies as well as mechanism elucidations. By understanding the current landscape and future prospects, the research community can identify opportunities for further development of energy‐dense SMB and potential integration with renewable energy systems.

## 2 Analysis of the Root Origins of the Na Dendrites

**Figure 2:**
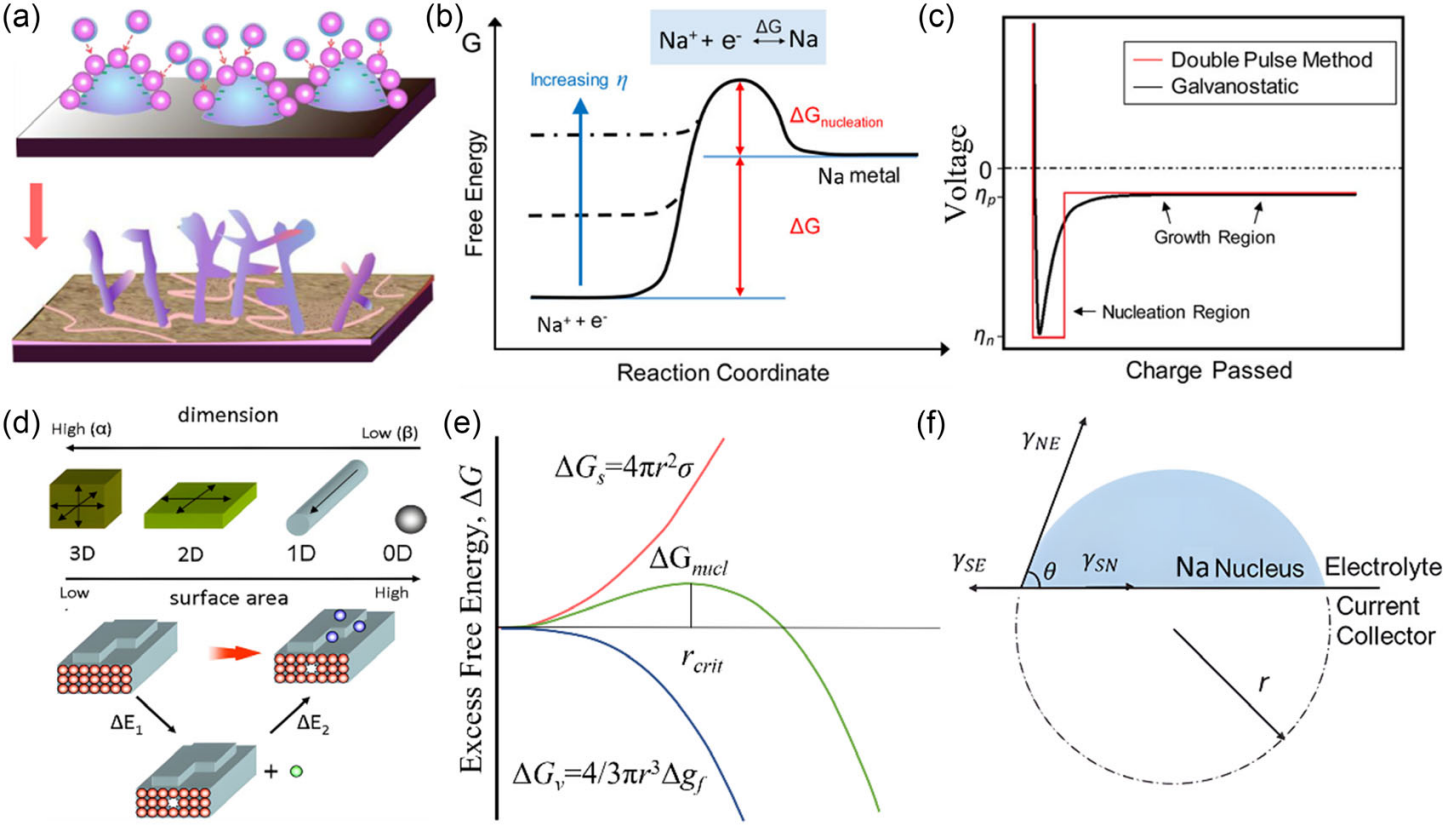
Nucleation driving force: a) Schematic diagram of the nucleation of Na electrodeposition on the surface of current collector;^[^ ^22^ ^]^ Copyright 2021, Wiley‐VCH. b) Free energy schematic showing the effects of increasing overpotential on the nucleation energy barrier;^[^ ^23^ ^]^ Copyright 2017, American Chemical Society. c) Schematic plot comparing the typical voltage profiles of galvanostatic Na deposition (black) and double pulse potentiostatic Na deposition (red);^[^ ^23^ ^]^ Copyright 2017, American Chemical Society. d) Scheme of high‐dimensional phases (α) and low‐dimensional phases (β);^[^ ^24^ ^]^ Copyright 2019, Elsevier. e) The dependence of the cluster free energy Δ*G* on the cluster radius according to the classical nucleation theory;^[^ ^24^, ^73^, ^74^ ^]^ Copyright 2012, Elsevier. f) Schematic diagram of a spherical sodium nucleus;^[^ ^25^ ^]^ Copyright 2015, Wiley‐VCH.

During the process of sodium deposition, the morphology of the initial nucleation plays a significant role in the subsequent growth behavior and cycling performance. In the initial stages of deposition, Na^+^ ions are reduced to Na atoms, which then aggregate to form small crystal nuclei, representing the nucleation process of sodium (**Figure** **2**a).^[^ ^22^ ^]^ Understanding the nucleation mechanism is crucial for regulating the growth behavior of sodium metal. Several models, based on theoretical simulations and experimental observations, have been proposed to provide the framework for explaining the nucleation process and offer insights into the factors that influence nucleation and subsequent growth.

### 2.1 Driving Force of Nucleation

Classical nucleation theory suggests that nucleation must overcome an initial free energy barrier. In electrodeposition, this barrier can be effectively controlled by adjusting the overpotential of the reduction reaction and manipulating the electrochemical supersaturation at the working electrode (Figure 2b).^[^ ^23^ ^]^ Electrocrystallization processes in traditional electrochemical systems are driven by various factors, which can be categorized into different types: reaction overpotential, charge transfer overpotential, diffusion overpotential, and surface/interface formation overpotential. Since it is challenging to separate and distinguish each source of polarization, the potential‐capacity curve of the sodium deposition process under constant current exhibits two notable characteristics: the nucleation overpotential (*η*_*n*_), represented by the voltage peak at the beginning of deposition, and the platform‐type overpotential (*η*_p_) during the sustained growth of sodium (Figure 2c).^[^ ^23^ ^]^ Specifically, at the beginning of the constant‐current sodium deposition process, the potential of the working electrode decreases below 0 V (*η*_*n*_). This electrochemical overpotential is sufficient to overcome the nucleation barrier and initiate the nucleation of sodium atoms. The nucleation overpotential arises from a combination of charge transfer overpotential and sodium/solution interface overpotential. Following the initial nucleation, the potential increases to *η*_p_, marking the start of the growth stage. The *η*_p_ overpotential primarily arises because Na^+^ ions need to traverse the solid electrolyte interface film to reach the surface of the electrode. Notably, the magnitude of |*η*_p_| is smaller than |*η*_*n*_| due to the lower energy barrier for further growth of existing crystal nuclei, which have already adsorbed sodium atoms, compared to the formation of new nuclei. This phenomenon contributes to a reduced overpotential during the growth stage (|*η*p| < |*η*n|). Furthermore, concentration polarization, influenced by the diffusion of electrolytes, contributes to the overpotential. Concentration polarization arises when the transport of ions in the electrolyte is hindered, leading to a difference in ion concentration near the electrode and resulting in additional overpotential.

### 2.2 Thermodynamics of Nucleation

Similar to lithium, sodium exhibits a relatively low surface energy, which makes it prone to the formation of one‐dimensional whisker structures and results in a higher specific surface area. Consequently, two thermodynamic factors, namely lower surface energy and higher migration energy, contribute to the growth of dendrites on the anode electrode made of sodium metal. Specifically, lower surface energy and a higher diffusion energy barrier are conducive to dendrite formation (Figure 2d).^[^ ^24^ ^]^ The nucleation process involves an increase in surface free energy and a decrease in volume free energy, and the stability of nucleation is determined by the overall change in free energy (Figure 2e).^[^ ^25^ ^]^ The classical nucleation Equation (1) can describe the relationship among nucleation size, overpotential, and current density. Assuming that the deposited crystal nucleus takes the form of a spherical crown with a radius of *r*, the Gibbs energy (Δ*G*_nucleation_) for the formation of a spherical crystal nucleus with a specific radius is the sum of the volume free energy and surface free energy (Figure 2f).^[^ ^25^ ^]^

(1) $$\Delta G_{\text{nucleation}}=-\frac{4}{3}\pi r^{3}\Delta G_{V}+4\pi r^{3}\gamma_{\text{NE}}Vm$$

Δ*G*_V_ —— the free energies with volume

γ_NE_ —— Surface energy at the nuclear/electrolyte interface

Depositional overpotential η is related to Δ*G*_V_

(2) $${\Delta G}_{V}\;=\;-\frac{F\left|\eta \right|}{V_{m}}$$

*η*—— Deposition overpotential

*F* ——Surface energy at the nuclear/electrolyte interface

*V*_m_ —— Molar volume of lithium

The critical radius is obtained as follows

(3) $$r_{\text{critical}}\;=\;-\frac{{2\gamma V}_{m}}{F\left|\eta \right|}$$

From Equation (3), it is evident that the size of the crystal nucleus is inversely proportional to the nucleation overpotential. The nucleation overpotential, in turn, is influenced by the deposition current. At high current density, the nucleation overpotential tends to be large, whereas at low current density, the overpotential decreases.

### 2.3 Nucleation Models

Three common models are commonly used to describe the nucleation process: the SEI‐induced nucleation model, the heterogeneous nucleation model, and the space charge model.^[^ ^25^ ^]^

#### 2.3.1 SEI‐Induced Nucleation Model

**Figure 3:**
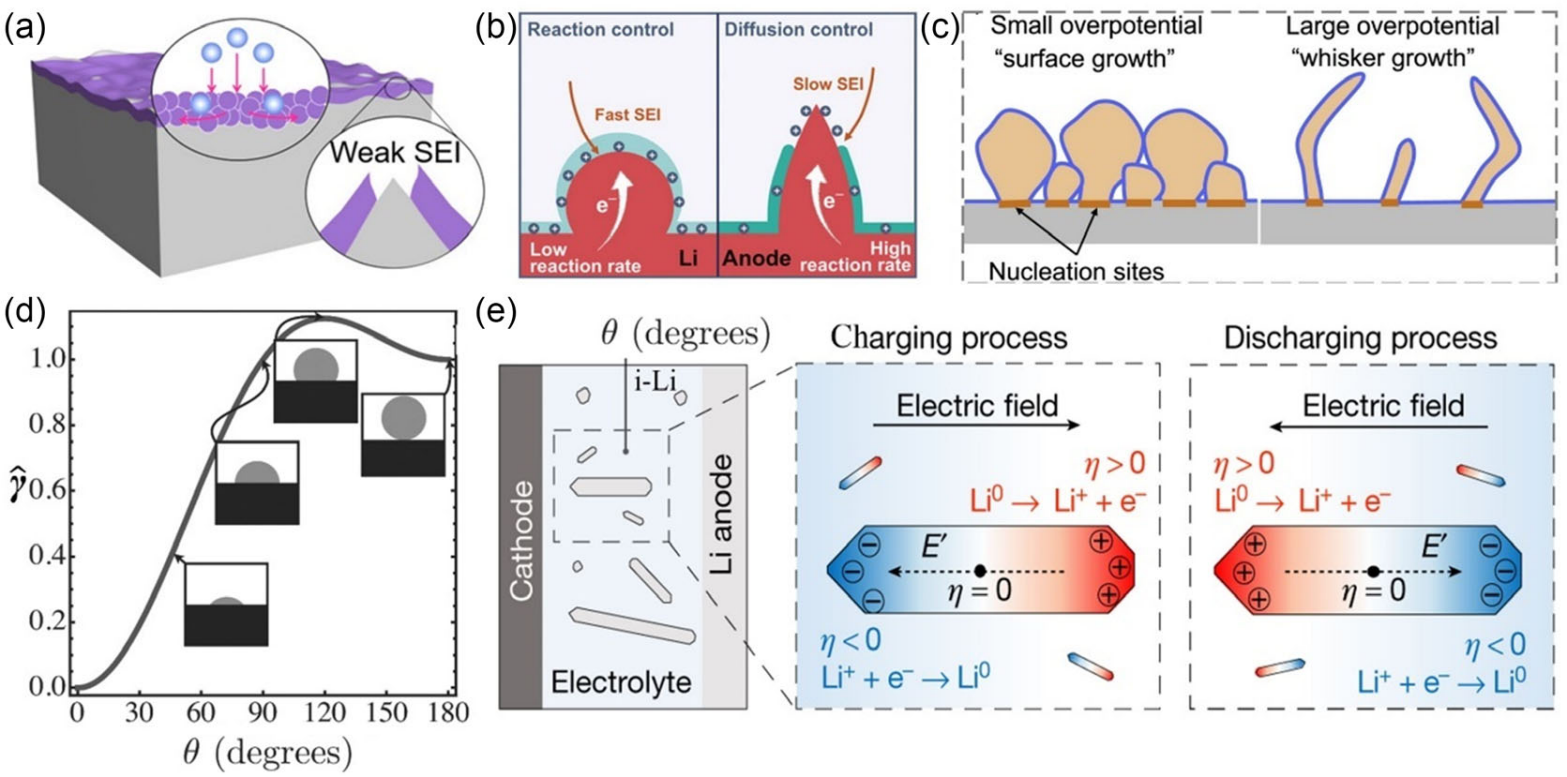
Nucleation mechanism of Na: a) Weak SEI on a Li anode prepared by soaking Li metal in LiPF_6_/EC/DEC electrolyte;^[^ ^21^ ^]^ Copyright 2023, Wiley‐VCH. b) The diffusion‐reaction competition mechanism causing spherical/dendritic Li deposition;^[^ ^26^ ^]^ Copyright 2020, Wiley‐VCH. c) Schematic illustration explaining root growth mechanism of lithium whiskers;^[^ ^27^ ^]^ Copyright 2017, Elsevier. d) Normalized surface energy, $\hat{\gamma }$ as a function of contact angle *θ*;^[^ ^28^ ^]^ Copyright 2023, The Electrochemical Society. e) Schematic illustration of the electric‐field‐induced charge separation on Li;^[^ ^29^ ^]^ Copyright 2021, Springer Nature. f) Profile of the ion concentrations *C*_c_, and *C*_a_ and electrostatic potential *V* resulting from the numerical simulation in the hypothetical case of uniform deposition with negligible growth of the cathode.^[^ ^30^ ^]^ Copyright 1990, American Physical Society.

The nonuniform compositional features of SEI layer would result in the localized concentration of ion influx toward the sodium metal electrode. Additionally, the fragile SEI film cannot bear with the significant volume expansion and mechanical stress upon cycling, thus generating the numerous cracks and electrochemical hot spots for the preferential sodium nucleation (**Figure** **3**a).^[^ ^21^ ^]^

In addition, the SEI is formed prior to sodium nucleation due to its low reduction potential, and the polycrystalline nature of the SEI film also contributes to sodium nucleation. Furthermore, random variations in the structure of the surrounding SEI can result in the dissolution of sodium dendrites at specific locations, leading to the formation of electrochemically inactive dead sodium. It should be noted that the SEI undergoes continuous collapse and reformation during repeated sodium stripping and plating processes, further amplifying the inhomogeneity of the SEI and adversely affecting sodium electrodeposition.

The composition of the SEI film on the sodium metal electrode is nonuniform, and the ionic conductivity is also uneven, leading to localized concentrations of electrons and ions. Moreover, due to the fragile nature of the SEI, the substantial volume expansion and mechanical stress during the process of sodium metal deposition and stripping result in numerous cracks in the SEI. These cracks act as electrochemically active sites, initiating sodium nucleation (Figure 3a).^[^ ^21^ ^]^

Additionally, owing to its low reduction potential, the SEI forms prior to the nucleation of sodium metal, and the polycrystalline nature of the SEI film also contributes to sodium nucleation. Furthermore, due to unpredictable alterations in the neighboring SEI structure, sodium dendrites might dissolve at their roots or particular locations, leading to the creation of electrochemically inactive sodium deposits. It is important to highlight that the SEI undergoes continual collapse and reorganization throughout repetitive sodium stripping and plating cycles. This behavior exacerbates the heterogeneity of the SEI and significantly impacts the electrodeposition of sodium in an adverse manner.

The growth of metal dendrites is governed by local interfacial dynamics, which includes solid‐phase diffusion in the SEI film. Specifically, the diffusion of metal ions within the SEI film is relatively swift under a small overpotential, leading to what is recognized as a dense surface growth pattern. In contrast, at higher magnitudes of overpotential, polarization experiences a significant rise, causing a notable reduction in the diffusion of metal ions within the SEI film. This results in metal deposition growing to a greater height, ultimately fostering dendrite formation (Figure 3b).^[^ ^26^ ^]^ The nucleation and growth of dendrites affected by the SEI can be divided into four stages: 1) In the first stage, spherical crystal nuclei appear on the surface of the negative sodium electrode, with their size proportional to the square root of the growth time. The SEI film passivates the surface of the sodium electrode and gradually reduces the rate of sodium deposition; 2) In the second stage, sodium dendrites grow from the roots and displace the initially formed sodium deposits away from the electrode.; 3) The third stage is characterized by a significant decrease in the growth rate due to the formation of a thickened SEI covering layer in the whisker portion of the dendrite; 4) In the fourth stage, whisker rupture occurs (Figure 3c).^[^ ^27^ ^]^ The continued slowdown in the initial surface growth rate may be attributed to the rapid formation of the SEI, which competes with the deposition of sodium and hinders Na^+^ transport. Additionally, SEI components with lower electron tunneling barriers also contribute to sodium nucleation. For instance, the Na_2_CO_3_/Na interface is less prone to stratification compared to the NaF/Na interface, but the Na_2_CO_3_/Na interface is more likely to induce nucleation and growth of sodium dendrites due to the presence of a smaller electron tunneling barrier.^[^ ^28^ ^]^

#### 2.3.2 Heterogeneous Nucleation Model

During the initial stage of deposition, the electron deposition of metal ions on the collector surface exhibits a heterogeneous nucleation behavior. This type of nucleation is influenced by various factors, including substrate properties, defects, impurities, and more. This type of nucleation is influenced by various factors, including substrate properties, defects, impurities, and more. The nucleation behavior is highly dependent on the characteristics of the substrate, such as its crystal structure, lattice matching, crystal plane orientation, and the presence of defects. A study conducted by Ely et al. employed numerical simulations to investigate the heterogeneous nucleation process, considering both thermodynamics and dynamics. They identified five distinct stages in this process, as depicted in Figure 3d.^[^ ^28^ ^]^ The first stage is nucleation inhibition, where the embryos formed are thermodynamically unstable and tend to redissolve back into the electrolyte. During the long incubation period, fluctuations in the electric and ion fields promote further growth of the embryos. Once the critical overpotential is surpassed, the embryos experience a short growth period, resulting in a narrow size distribution. Finally, the embryos with the critical kinetic radius undergo rapid growth with increasing overpotential until they reach a state of thermodynamic and kinetic stability. In this state, they maintain a constant growth rate until the final formation is achieved. The relation between contact angle and critical free energy can be obtained from heterogeneous nucleation theory. The critical free energy change (${\Delta G}_{\text{eq}}^{*}$) is given.

(4) $$\text{cos}\theta \;=\;-\frac{\gamma_{\text{NE}}-\gamma_{\text{SE}}}{\gamma_{\text{NE}}}$$

(5) $${\Delta G}_{\text{eq}}^{*}\;=\;\frac{1}{3}{(4\pi r}_{\text{eq}}^{*}{2)\gamma }_{\text{SN}}(\frac{2-{3\text{cos}\theta \;+\text{cos}}^{3}\theta }{4})$$

θ —— Contact Angle between nucleus and electrolyte

$\gamma_{\text{SE}}$ ——substrate /Interfacial free energy of electrolyte

$\gamma_{\text{SN}}$ ——substrate /Interfacial free energy of nucleus

$\gamma_{\text{NE}}$ ——nucleus/ Interfacial free energy of electrolyte

#### 2.3.3 Space Charge Model

After the nucleation of sodium, the Na^+^ and anions disperse and migrate toward the negative and positive electrodes, respectively. This migration results in a decrease in anion concentration on the negative electrode surface and the formation of a space charge and electric field near the electrode/electrolyte interface, which triggers dendrite growth. At high current densities, an excess positive charge leads to localized space charge and an associated electric field, making the negative electrode more susceptible to issues such as uneven deposition and the formation of dendritic crystals (Figure 3e).^[^ ^29^ ^]^ To explain the nucleation of dendritic metals, such as lithium, induced by the space charge effect, Chazalviel conducted calculations on the distribution of electrostatic potential and ion concentration in a symmetric cell. This equation describes the dynamic behavior of ion concentration in the presence of diffusion and electric field effects, providing insights into how the space charge influences dendrite growth.

(6) $$\frac{\partial C}{\partial x}(x\;=\;0)\;=J/eD(1+\frac{\mu_{c}}{\mu_{a}})$$

J —— Current density

*e* —— Electron charge

D —— Diffusion coefficient

$\mu_{c}$/ $\mu_{a}$——Number of migrations of anions/cations

According to Formula (6), the behavior of metal deposition can be predicted based on the initial concentration of Na^+^ (C_0_), diffusion coefficient (*D*), and effective current density (*J*) in the electrolyte. When the concentration gradient ∂C/∂x < ${2C}_{0}/L$, where *L* is the characteristic length, the ion concentration in the electrolyte reaches a steady state and the electrodeposition exhibits a flat morphology. However, when the concentration gradient ∂C/∂x > ${2C}_{0}/L$, there is a significant difference in ion concentration between the surfaces of the positive and negative electrodes. The ion concentration in the negative microregion rapidly decreases to zero, creating an electronegative space. This leads to a significant increase in polarization voltage, promoting dendrite growth (Figure 3f).^[^ ^30^ ^]^ When the concentration of Na^+^ on the electrode surface reaches zero, dendrites start to form. The initial time of dendrite growth is referred to as Sand's time (*τ*), which can be expressed as
(7)

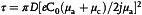




Indeed, the transition time “τ” is directly proportional to the concentration of sodium salt (*C*_0_), and inversely proportional to the effective current density (*J*). This implies that a lower initial concentration of sodium salt and a higher effective current density on the electrode surface increase the likelihood of dendrite formation. The effective current density *J* influences the behavior of the ion concentration gradient, and the limiting current density J* can be expressed as follows

(8) $$J*\;=\;\frac{{2eDC}_{0}{(\mu }_{a}{\;+\;\mu }_{c})}{{L\mu }_{a}}$$

At low effective current density (*J* < J*), the ion concentration gradient remains minimal and stable, preventing the formation of dendrites. However, at high effective current density (*J* > J*), the ion concentration in the microregion of the negative surface decreases to zero, resulting in uneven nucleation and the growth of dendrites. Therefore, reducing the effective current density or optimizing the distribution of the electric field can help inhibit dendrite formation. Furthermore, ion transport in the electrolyte is primarily governed by diffusion, while ion transfer in the space charge region is influenced by electric field migration, where the potential is less than 0 V. The uneven distribution of ion flow leads to the formation of crystal nuclei on the surface of sodium, and the protruding tips tend to accumulate electrons and exhibit a high concentration of local electric field, thereby inducing ion aggregation and deposition, ultimately resulting in dendrite formation.

In addition to the basic factors brought by electrolytes, electrodes, and the SEI layer, external conditions such as current density, charging capacity, operating temperature, and internal pressure also significantly affect the growth rate and growth pattern of dendrites. It is commonly observed that high current density and prolonged charging time result in more pronounced dendrite growth. However, when the sodium deposition is charge transfer controlled and the current is relatively small, the deposition can also be well distributed as the current density increases. The empirical formula and corresponding curve of the influence of charge amount and current density on electrode surface morphology can be expressed as. It is generally believed that high current density and long charging time lead to severe dendrite growth. However, in cases where sodium deposition is primarily controlled by charge transfer and the current is relatively low, an increase in current density can actually lead to a well‐distributed deposition. The relationship between the charge amount, current density, and electrode surface morphology can be described by empirical formulas and corresponding curves.^[^ ^31^ ^]^

(9) $$Q\;=\;\frac{5.58133}{1-1.0286J\;+\;0{\text{.4957}J}^{2}}$$

(10) $$\left\{{\left(J-\text{1}\right)}^{2}\;+\;1\right\}Q\;\approx \;11$$

*J* —— Current density

*Q* —— Total charge

**Figure 4:**
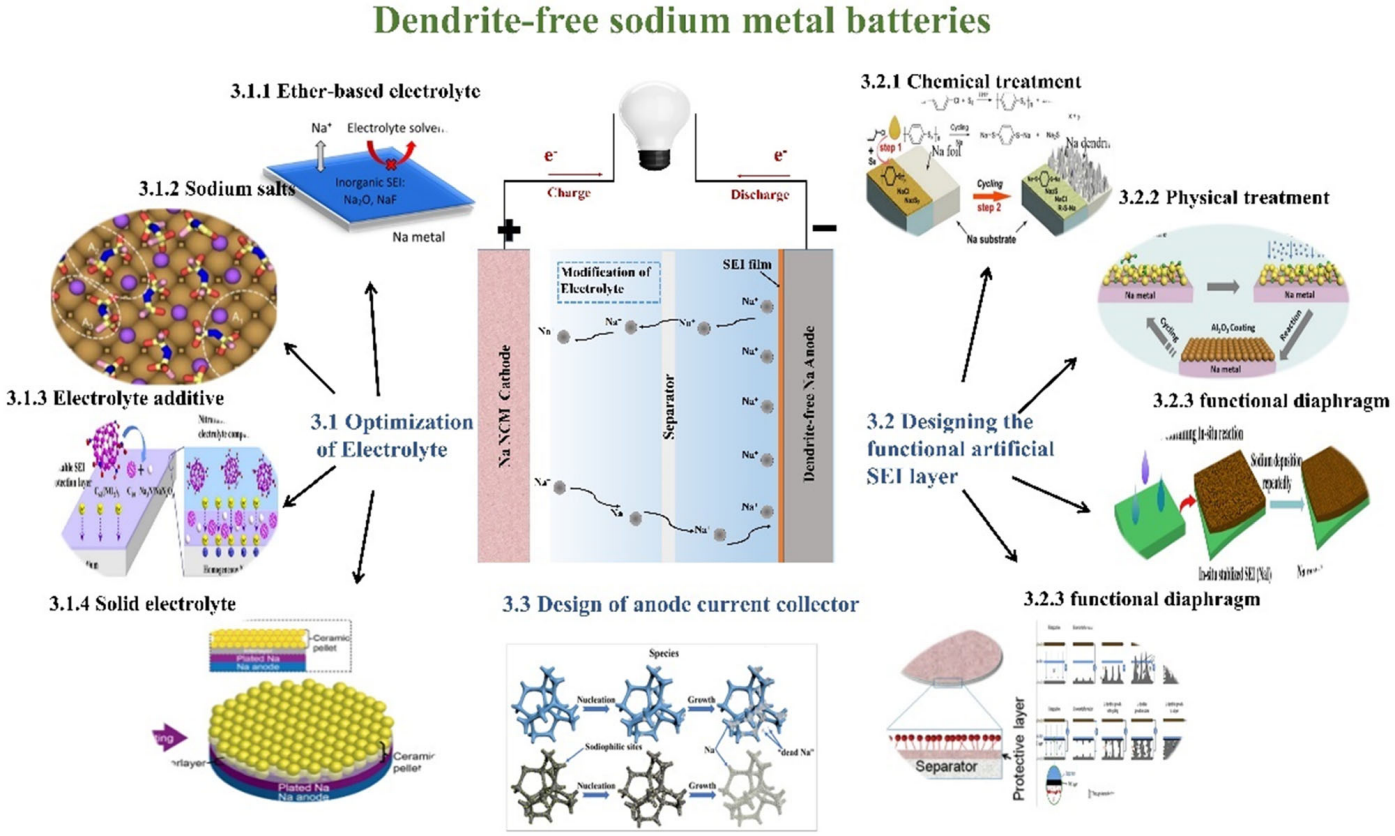
Dendrite‐free Na metal anode modification strategy.^[^ ^5^, ^6^, ^14^, ^15^, ^16^, ^32^, ^34^, ^75^, ^76^, ^77^, ^78^, ^79^, ^80^, ^81^, ^82^, ^83^, ^84^, ^85^, ^86^, ^87^ ^]^

According to the aforementioned failure mechanism, controlling sodium metal deposition behavior, optimizing the SEI layer, and managing sodium activity and volume change are crucial for achieving ideal electrochemical performance and securing the reliable operation of the cell model, particularly in the context of research focusing on energy‐dense cell models, emphasizing the criticality of multidimensional and delicate regulation for the metallic deposition behavior **Figure** **4**.^[^ ^32^ ^]^

## 3 Optimization Strategies of the Cation Utilization Degree

### 3.1 Electrolyte Modification

SEI plays a crucial role in ensuring the cyclic stability of SMBs.^[^ ^33^ ^]^ Researchers have long aimed to achieve an ideal SEI with excellent chemical and electrochemical stability, as well as mechanical stiffness. During the repetitive charge and discharge cycles, the SEI components are formed through the decomposition of electrolyte constituents.^[^ ^34^ ^]^ Therefore, by carefully controlling the electrolyte composition, concentration, and incorporating suitable additives, the SEI components on the sodium metal electrode surface can be enhanced, resulting in controlled Na^+^ deposition and effective suppression of dendritic growth. To facilitate the electrochemical stability of SMBs, the electrolyte should meet the following criteria: 1) Thermodynamic and kinetic stability toward the sodium metal anode, allowing for the formation of a robust and stable SEI with high coulombic efficiency; 2) Compatibility with the working voltage of the positive electrode material, ensuring the stability and noncorrosiveness of the electrolyte; 3) Ensuring safety by promoting uniform deposition of Na^+^. By considering these conditions, researchers have made significant advancements in achieving dendrite‐free SMBs that exhibit favorable Na^+^ utilization.

#### 3.1.1 Ether‐Based Electrolyte

Electrochemical decomposition, encompassing reduction and oxidation, correlates with the electronic states of solvents and solvated cations under an applied potential. This relationship is linked to the energy levels of the relatively lowest unoccupied molecular orbital (LUMO) and the highest occupied molecular orbital (HOMO). In accordance with Goodenough's model, the electrochemical stability window of an electrolyte can be deduced from the relative disparity between its HOMO and LUMO values.^[^ ^35^ ^]^ In comparison, the LUMO demonstrates an electron‐acceptor characteristic due to its strong affinity for electrons. Consequently, a higher HOMO level signifies easier electron loss, implying a weaker oxidative stability of the molecule. Conversely, a higher LUMO level indicates a more challenging electron acquisition, suggesting a heightened reductive stability of the molecule.

**Figure 5:**
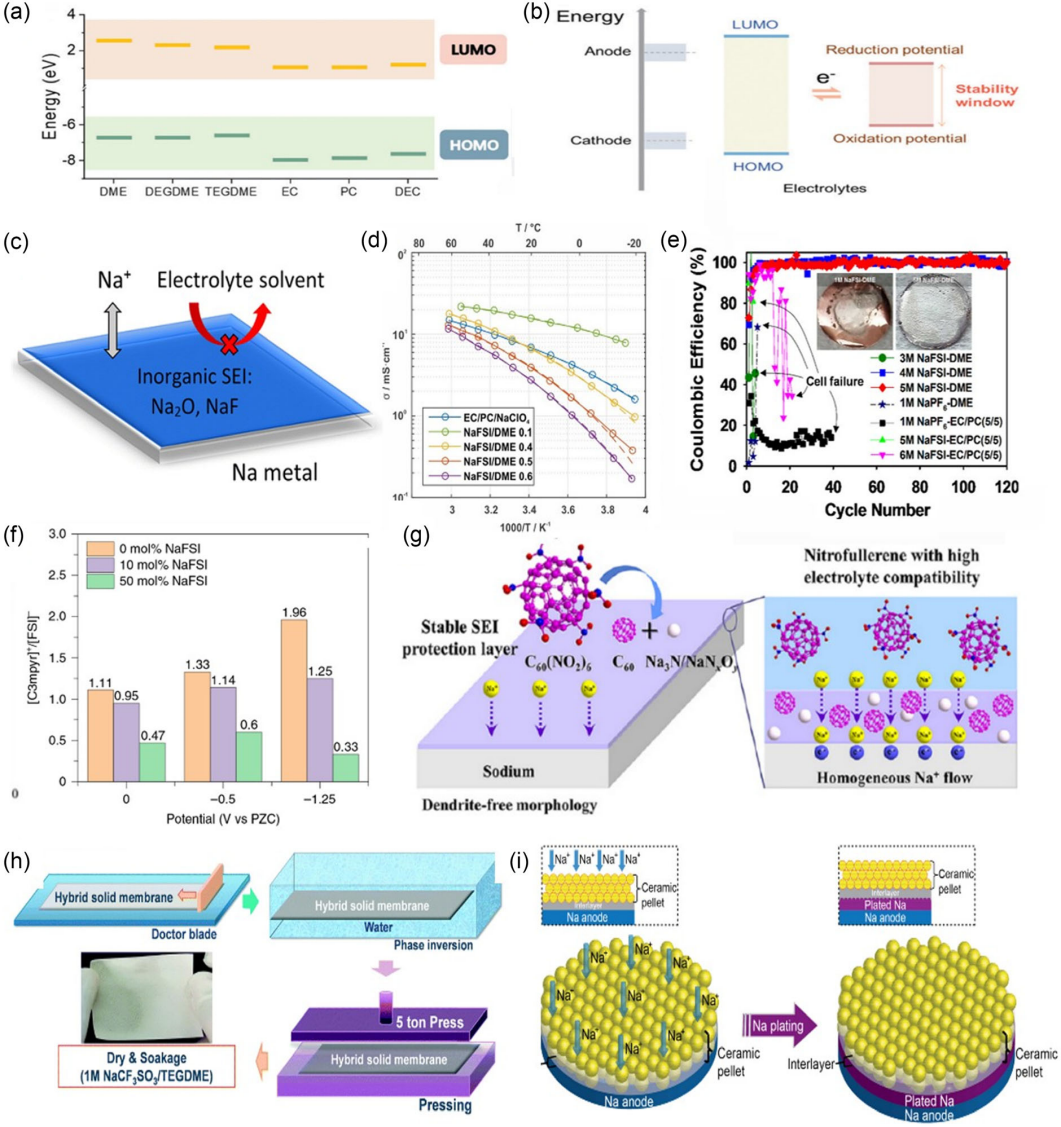
Electrolyte modification strategies: a) LUMO and HOMO levels of ether solvents (DME, DEGDME, and TEGDME) and carbonate solvents (EC, DEC, and PC);^[^ ^37^, ^88^ ^]^ Copyright 2018, Wiley‐VCH. b) Relation between the HOMO/LUMO levels and the electrolyte stability window;^[^ ^88^ ^]^ Copyright 2018, Wiley‐VCH. c) Schematic showing the SEI formed on the Na metal surface using NaPF_6_ in glymes;^[^ ^38^ ^]^ Copyright 2015, American Chemical Society. d) Specific ionic conductivity versus inverse temperature for 1 m NaClO_4_ in EC/PC (1:1 m m^−1^) and NaFSI/DME electrolytes;^[^ ^39^ ^]^ Copyright 2016, John Wiley & Sons. e) Coulombic efficiency for Na plating/stripping in Na/SS cells in of NaFSI‐DME;^[^ ^40^ ^]^ Copyright 2017, American Chemical Society. f) Ratio of [C_3_mpyr]^+^ to [FSI]^−^ number and number of Na^+^ versus PZC for C_3_mpyrFSI with 0, 10 and 50 mol% NaFSI;^[^ ^43^ ^]^ Copyright 2020, Springer Nature. g) Schematic illustration of the effect of C_60_(NO_2_)_6_ additive on the Na deposition behavior;^[^ ^44^ ^]^ Copyright 2021, Elsevier. h) HSE hybrid preparation process, involving NASICON powder, PVdF‐HFP, and 1 M NaCF_3_SO_3_/TEGDME;^[^ ^51^ ^]^ Copyright 2015,Royal Society of Chemistry. i) Schematic illustration of Na plating using polymer film as an interlayer between Na_3_Zr_2_(PO_4_)(SiO_4_)_2_ ceramic electrolyte and Na metal.^[^ ^52^ ^]^ Copyright 2017, American Chemical Society.

Compared to ether‐based electrolytes, carbonate electrolytes are more susceptible to react with sodium metal, resulting in the formation of fragile SEI layers. This phenomenon contributes to severe safety issues, including dendrite formation and reduced coulombic efficiency. In contrast, ether‐based electrolytes, with their higher LUMO, tend to promote the decomposition of sodium salts rather than solvents.^[^ ^36^ ^]^ **Figure** **5**a,b discusses the HOMO/LUMO levels of distinct solvents (ethers and carbonates) and various solvated‐Na^+^. Among individual solvents, ether solvents such as glycol dimethyl ether (DME), DEGDME, and TEGDME consistently exhibit higher LUMO levels and elevated HOMO values compared to carbonate solvents (EC, PC, and DEC).^[^ ^37^ ^]^ This starkly signifies the superior reductive stability and comparatively lesser oxidative stability of ether solvents in contrast to conventional carbonate solvents. The pronounced reductive stability of ether solvents indicates their reduced likelihood of decomposing during the discharging process, which concurrently contributes to the development of a thin SEI layer.

In the work conducted by Cui et al., the electrolyte system of NaPF_6_‐diglyme was employed. This electrolyte facilitated the formation of a thin and uniform inorganic SEI film consisting of NaF and Na_2_O on the surface of the sodium metal electrode. This film effectively prevents further reaction between sodium metal and the electrolyte, promotes homogeneous Na^+^ ion flux, and successfully inhibits dendrite formation. Notably, long‐term dendrite‐free sodium deposition and stripping were achieved using this electrolyte, exhibiting an average coulombic efficiency of 99.9% over 300 cycles at a current density of 0.5 mA cm^−2^ and areal capacity of 1 mA cm^−2^ (Figure 5a).^[^ ^38^ ^]^

Additionally, solvents such as ethylene glycol dimethyl ether (DME) and tetrahydrofuran (THF) have shown the ability to form stable SEI layers on sodium metal surfaces (Figure 5b,c).^[^ ^39^, ^40^ ^]^ DME, with its high LUMO energy level, exhibits good compatibility with sodium metal. When combined with high concentrations of sodium salts, a robust SEI film is formed on the surface of the sodium metal. The robust structure of the SEI film is crucial for the efficient utilization of Na^+^.^[^ ^41^ ^]^

In conclusion, the selection of appropriate electrolytes, especially those based on ether solvents, plays a critical role in achieving stable and dendrite‐free sodium metal anodes in advanced battery systems.

#### 3.1.2 Sodium Salts

Sodium salts play a crucial role in the formation of the SEI in ether‐based electrolytes. The presence of different sodium salts leads to the formation of SEI layers with varying compositions, thicknesses, and structures. The concentration of the salt in the electrolyte plays a significant role in determining the performance of the electrolyte and the stability of the electrode/electrolyte interface. Highly concentrated electrolytes offer several advantages, including minimizing side reactions, increasing carrier density, widening voltage windows, enhancing safety, and extending the lifespan of the battery.^[^ ^42^ ^]^

Chen et al. conducted a study using atomic force microscopy and molecular dynamics simulation to investigate the influence of NaFSI salt concentration and applied potential on the chemistry of the electrolyte interface. The study revealed that the interface structure of the superconcentrated 1‐methyl‐1‐propyl pyrroline bis (fluoro‐sulfonyl) imide (C_3_mpyrFSI) ionic liquid (IL) containing 50 mol% NaFSI differed significantly from that of pure ionic liquid or its electrolyte with low salt concentration. This research highlights the potential benefits of designing low melting point electrolytes or new anionic/ionic liquid solvents to enhance high salt solubility (Figure 5e).^[^ ^43^ ^]^

#### 3.1.3 Electrolyte Additive

The inclusion of electrolyte additives serves as a mean to stabilize the SEI and enhance the cyclic performance of batteries. Commonly used electrolyte additives, such as 1,3‐propane sultone (PS), vinylene carbonate (VC), fluoroethylene carbonate (FEC), and nitrofullerene (C_60_(NO_2_)_6_) play a significant role in improving the stability of the SEI and overall battery performance. Cheng et al. synthesized the additive nitrofullerene C_60_(NO_2_)_6_ by introducing nitro groups to the fullerene (C_60_) structure. This addition greatly facilitated the formation of a stable SEI on the negative surface of sodium metal, ensuring uniform sodium deposition and effectively inhibiting the growth of sodium dendrites. The results demonstrated that the inclusion of C_60_(NO_2_)_6_ in carbonate and ether battery electrolytes resulted in low voltage hysteresis, high performance, and long cycle life, indicating the beneficial impact of this additive (Figure 5f).^[^ ^44^ ^]^

#### 3.1.4 Solid‐State Electrolyte

Solid‐state electrolytes in sodium batteries can serve a dual function as electrolytes and artificial SEI films on the negative electrode. Currently, solid‐state electrolytes can be classified into three main types: ISEs, PSEs, and CSEs.^[^ ^45^ ^]^

ISEs stand out among various solid‐state electrolytes due to their exceptional thermal stability, high ionic conductivity, and nonflammability. Thanks to their robust covalently or ionically bonded framework, inorganic solid‐state electrolytes can function effectively across an extensive temperature range, while liquid electrolytes and polymer solid‐state electrolytes would experience freezing or decomposition. Inorganic electrolyte materials such as β‐Al_2_O_3_, Na superconductors (NASICON), sulfides, and complex hydrides exhibit high ionic conductivity and good flame‐retardant properties. However, they often lack interfacial stability and compatibility.^[^ ^46^ ^]^

PSEs offer favorable processability and interface compatibility.^[^ ^47^ ^]^ These electrolytes can be prepared by dissolving metal salts within a polymer matrix characterized by a relatively high molecular weight.^[^ ^48^ ^]^ In the course of charging and discharging a battery, sodium ions become solvated by the polymer chain and move along its molecular chain. Nevertheless, the mobility of sodium ions along the polymer chain is significantly influenced by temperature, leading to a reduced rate of sodium ion migration within the polymer solid electrolyte at room temperature. Most polymer solid electrolytes demonstrate satisfactory rates of sodium ion migration only at elevated temperatures, typically above 80 °C. Among the polymer matrix commonly employed in polymer solid electrolytes are polyethylene oxide (PEO), polymethyl methacrylate (PMMA), polyvinylidene fluoride (PVDF), polyvinylidene fluoride with hexafluoropropylene (PVDF‐HFP), polyvinyl chloride (PVC), polyacrylonitrile (PAN), and polyvinyl alcohol (PVA). PEO stands out as the most widely utilized polymer due to its supple ethylene oxide segments and ether oxygen atoms.

In contrast to PSEs, CSEs exhibit exceptional mechanical properties and high ionic conductivity, thereby effectively inhibiting dendrite growth. While these polymers have relatively low ionic conductivity at room temperature, incorporating inorganic fillers into the polymer matrix offers a promising solution to address the challenges of dendrite formation and low conductivity.^[^ ^49^ ^]^ Inorganic fillers can be classified as inactive ceramic powders (TiO_2_, SiO_2_, ZrO_2_, AlCl_3_) and ionic conductors (NASICON). The addition of inactive ceramic powders improves the mechanical properties of the electrolyte while slightly increasing interfacial resistance. More recently, the use of PEO/Na_2_Zn_2_TeO_6_ (PEO/NZTO) as an electrolyte has shown superior cyclic stability at low polarization voltage. This electrolyte exhibits low sodium/electrolyte interface resistance and excellent stability against the metal sodium anode.^[^ ^50^ ^]^ Kim et al. reported a NASICON‐based composite solid electrolyte that offers high safety and meets the required properties of SMBs, including high ionic conductivity, a wide electrochemical window, low solid/solid interface resistance, and high thermal stability (Figure 5h).^[^ ^51^ ^]^ Additionally, a smooth and dendrite‐free sodium metal negative electrode can be achieved by coating the PEA/NZSP/PVDF‐HFP electrolyte (Figure 5i).^[^ ^52^ ^]^

### 3.2 Designing the Functional Artificial SEI Layer

Establishing a stable artificial SEI film holds paramount significance in ensuring the security of SMBs, necessitating a comprehensive understanding of the role played by SEI on the surface of SMBs. Numerous investigations have been undertaken to develop effective SEI membranes, primarily involving the utilization of ether‐based electrolyte, sodium salts, electrolyte additives, or solid‐state electrolytes. While the implementation of these strategies has yielded certain favorable outcomes in constructing uniform and stable SEI, in most instances, the additives used for SEI film formation are continuously consumed and struggle to withstand long‐term sodium plating/peeling, particularly within confined spatial constraints.

To overcome this challenge, the development of an artificial SEI protective layer has emerged as a promising approach. This protective layer is specifically designed to prevent undesirable side reactions between the electrolyte and sodium metal anode. In comparison to the spontaneously formed SEI layer, the artificial SEI layer offers several advantages. Firstly, it allows for precise control over its thickness, ensuring a uniform surface and exhibiting a dense structure. Furthermore, this artificial interface layer serves to avert direct contact between Na metal and the liquid electrolyte, effectively curbing undue electrolyte and electrode consumption while also preventing dendrite formation. This, in turn, fosters the attainment of uniform Na deposition. Through an amalgamation of research on chemical treatment, physical manipulation, and functional membrane fabrication for artificial interface layers, we delineate their pivotal role within the sodium plating/stripping process for SMBs.^[^ ^33^, ^53^, ^54^, ^55^ ^]^

#### 3.2.1 Chemical Treatment

**Figure 6:**
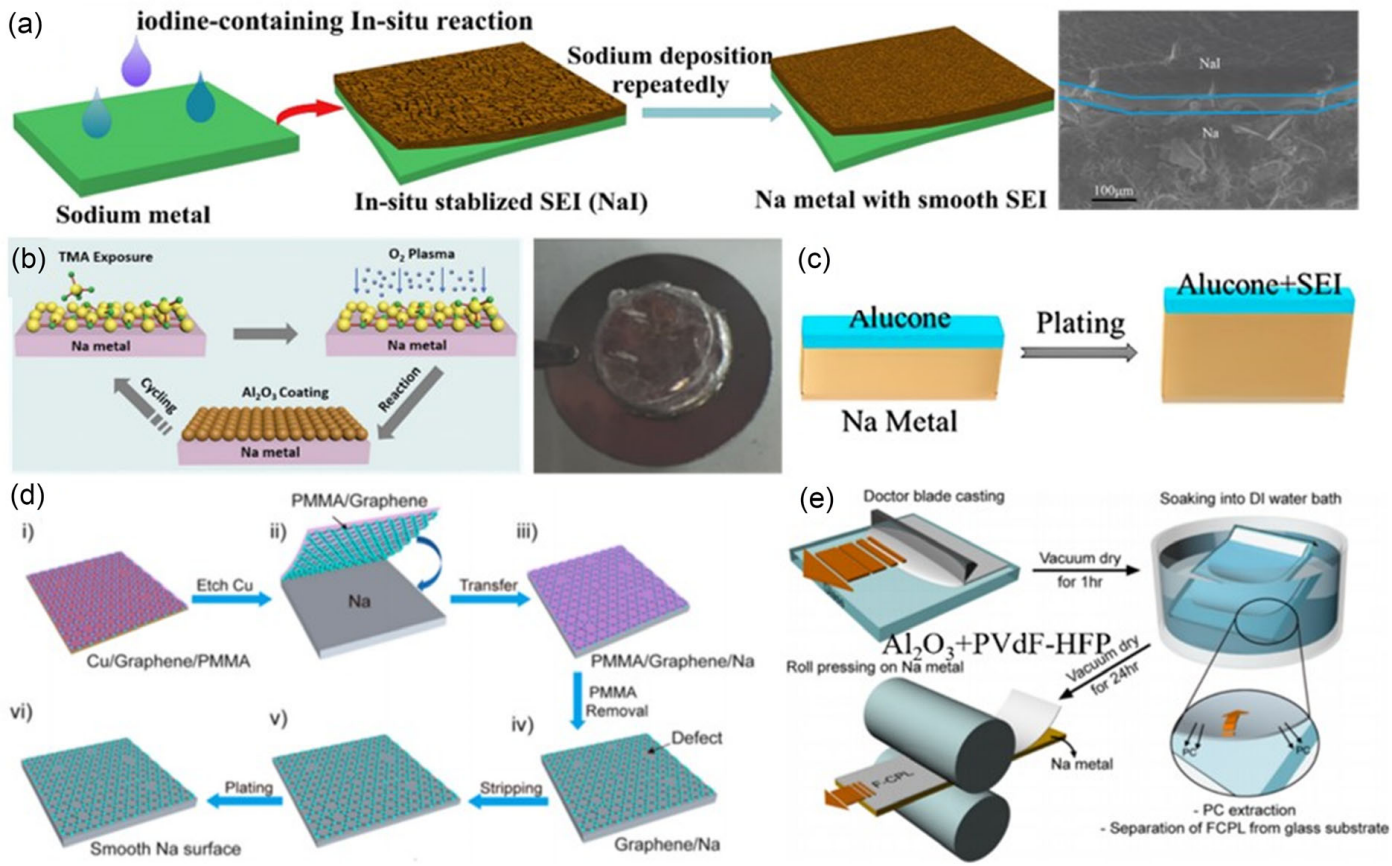
Construct various artificial SEI layers: a) Schematic illustration of the preparation of NaI‐coated Na and its cyclic stability, and SEM image of metal Na anode after treatment by 2‐iodopropane;^[^ ^58^ ^]^ Copyright 2019, Elsevier. b) Schematic showing a stable Al_2_O_3_ coating and its function on Na plating, and photographs of Na@Al_2_O_3_;^[^ ^61^ ^]^ Copyright 2016, Wiley‐VCH. c) Schematic diagrams of Na with MLD alucone coating;^[^ ^62^ ^]^ Copyright 2017, American Chemical Society. d) Illustration of transferring free‐standing graphene film onto Na metal surface, and the high stability of graphene‐coated Na anode during stripping/plating without the formation of Na dendrites;^[^ ^63^ ^]^ Copyright 2017, American Chemical Society. e) Schematic of the Al_2_O_3_ + PVDF‐HFPF/Na metal electrode integration fabrication.^[^ ^65^ ^]^ Copyright 2017, American Chemical Society.

The preparation of a robust artificial SEI layer through reactions with sodium metal solutions has proven to be effective in inhibiting dendrite formation and extending the service life of SMBs. For instance, a low‐cost bismuth (Bi) film generated on metallic sodium by a simple substitution reaction has been shown to exhibit high exchange currents and rapid charge transfer kinetics, resulting in uniform sodium deposition.^[^ ^56^, ^57^ ^]^ Wang demonstrated the in situ formation of a NaI SEI layer based on a conversion chemical reaction, which was further validated through experiments and DFT calculations. The results showed that NaI had lower Na^+^ diffusion barriers compared to NaF, effectively stabilizing the uniform deposition of sodium (**Figure** **6**b).^[^ ^58^ ^]^ Another approach, developed by Archer, involved the electrochemical polymerization process to construct an imidazole‐type ionic liquid (IL) functional film on the sodium metal anode as the SEI layer. During charging, the monomer received electrons from the sodium metal, forming a reactive substance that reacted with additional monomers to create active centers. Polymerization then occurred, resulting in the formation of a thin layer on the surface of the sodium metal cathode. Optical microscope observation demonstrated that the artificial film effectively stabilized the sodium metal deposition layer without dendrite formation, and the overall battery exhibited a coulombic efficiency of 100% over 100 cycles, matching the positive electrode of Na_3_V_2_(PO_4_)_3_.^[^ ^59^ ^]^

These examples serve as compelling evidence for the potential of artificial SEI layers in promoting uniform sodium deposition, inhibiting dendrite growth, and achieving high performance and stability in SMBs.

#### 3.2.2 Physical Treatment‐Coating Protection‐ALD

The advancement of film preparation technology has led to the exploration and extensive research on physical coatings for SMBs.

Among these techniques, atomic layer deposition (ALD) stands out as a method capable of precise control over coating thickness and uniformity.^[^ ^60^ ^]^ Hu, for instance, employed low‐temperature plasma‐enhanced atomic layer deposition (PEALD) to create an artificial SEI layer on the surface of sodium metal. A 2.8 nm Al_2_O_3_ film was deposited, providing protection against electrolyte decomposition and inhibiting dendrite formation. This resulted in significantly enhanced cycle stability even after 500 cycles at 3 mA cm^−2^ (Figure 6c).^[^ ^61^ ^]^ Similarly, Sun et al. utilized trimethyl aluminum (TMA) and ethylene glycol (EG) through continuous action deposition at 85 °C to obtain an advanced molecular layer deposition called alucone. This coating effectively suppressed the formation of dendrites and mossy‐like sodium, thereby significantly improving the battery's lifespan (Figure 6d).^[^ ^62^ ^]^

These examples demonstrate the potential of physical coating techniques, such as ALD and molecular layer deposition, in creating artificial SEI layers that offer improved stability, dendrite inhibition, and extended cycle life in SMBs.

#### 3.2.3 Functional Diaphragm

Apart from preassembly treatments, another approach to enhance the electrochemical performance of the sodium metal anode is by applying an independent protective film in a scalable and cost‐effective manner. Li conducted a study where they directly coated the sodium metal surface with a self‐supported graphene film of adjustable thickness. The dependence of sodium anode stability on graphene film thickness was systematically investigated under different current densities and capacities. Remarkably, a sodium anode with a 5 nm multilayer graphene protective layer achieved stable cycling behavior in a carbonate electrolyte without any additives, marking a significant breakthrough (Figure 6e).^[^ ^63^ ^]^

Li also proposed the use of a thin commercial carbon cloth to cover the sodium metal anode as a protective layer, leveraging its large surface area to dissipate current density. The protected metallic sodium anode exhibited superior cyclic stability in carbonate and ether electrolytes at various current densities.^[^ ^64^ ^]^

Furthermore, a simple method of forming a protective layer on the surface of the sodium negative electrode was reported. It involved the combination of Al_2_O_3_ particles and vinylidene fluoro‐hexafluoropropylene, forming an inorganic‐organic composite protective layer that mechanically inhibits the growth of sodium dendrites (Figure 6f).^[^ ^65^ ^]^

The inorganic–organic composite layer serves multiple functions in stabilizing the sodium metal negative electrode: the inorganic component mechanically inhibits dendrite growth, while the organic component reduces electrolyte decomposition, enhances ionic conductivity, and provides a flexible surface for uniform deposition and stripping. These studies have significantly improved the stability of the sodium metal anode and inhibited dendrite growth. However, further research on the integration of these protective layers into the whole battery system is necessary to fully explore their potential benefits.

### 3.3 Structural Design of the Electrode

The deposition process of sodium metal anodes can be influenced by various factors, including current density, cation dispersion, and the nature of the collector material. Lower current densities and better cation dispersion are known to promote the deposition of dendrite‐free sodium anodes, in accordance with Sand's law and the metal nucleation mechanism.

In contrast to conventional two‐dimensional collectors, three‐dimensional (3D) skeletons offer several advantages in the context of sodium metal anodes. The increased surface area of the 3D structure promotes the dispersion of current density, effectively delaying dendrite growth. This characteristic is beneficial for achieving a stable anode. Additionally, a sodiophilic 3D skeleton facilitates the high dispersion of Na^+^ ions, leading to uniform metal deposition. The unique structure of the 3D skeleton also helps to limit the volume expansion of sodium during cycling, thereby enhancing the stability of the SEI formed on the anode.^[^ ^66^ ^]^

From a device perspective, incorporating 3D porous materials into the sodium metal anode can reduce the battery's weight, thereby increasing its energy density.

There are two main approaches to realizing a 3D composite sodium metal anode. The first method involves fusing sodium metal into the 3D skeleton through a melting process, resulting in a composite electrode. The second method entails depositing sodium metal uniformly onto the surface of a 3D collector, forming a composite negative electrode.

Furthermore, 3D skeletons can be categorized into two main types based on the material used: metal‐based and carbon‐based. Each type has its own action mechanisms and advantages. The specific characteristics and benefits of these different skeleton systems will depend on the choice of material and its sodiophilic properties.

#### 3.3.1 Metal 3D Skeleton

Indeed, researchers have explored various 3D copper‐based collectors as alternatives to traditional copper foil for SMBs. These include 3D copper, porous copper, copper mesh, and columnar copper structures. By replacing the conventional copper foil, these 3D copper collectors offer advantages such as increased surface area, improved nucleation sites, enhanced diffusion channels for Na^+^, and reduced local current density. These features contribute to achieving controlled and dendrite‐free sodium deposition/stripping.^[^ ^67^, ^68^ ^]^

**Figure 7:**
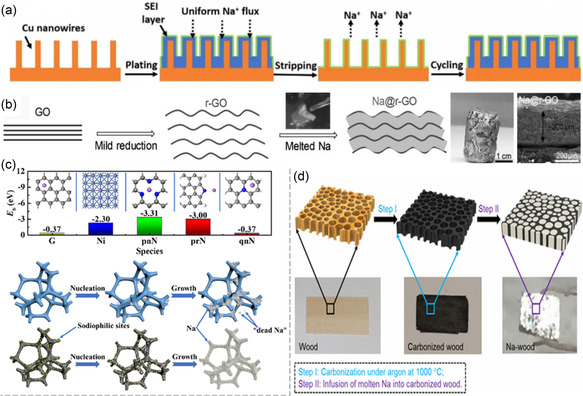
Construction of metal current collector: a) Cu nanowires in situ formed on Cu foam can provide more nucleation sites and effectively limit the final size of deposited Na, this is beneficial for reducing local current density, stabilizing the SEI layer, and suppressing the dendrites growth.^[^ ^69^ ^]^ b) Construction of 3D carbon current collector: Schematic representation of the preparation of Na@RGO composites, SEM images, and optical photograph of the composites;^[^ ^71^ ^]^ Copyright 2017, Wiley‐VCH. c) Binding energy of a Na atom with graphene (G), Ni, pyridinic N, pyrrolic N, and quaternary N of NG, with insets showing top views of optimized structures of Na on NG;^[^ ^70^ ^]^ Copyright 2020, American Chemical Society. d) Encapsulation of metallic sodium into carbonized wood by a spontaneous and instantaneous infusion.^[^ ^72^ ^]^ Copyright 2017, American Chemical Society.

Fan et al. proposed a surface modification method for copper collectors, where copper nanowires were formed in situ on 3D porous copper foam. This approach significantly increased the surface area of the copper foam, providing abundant nucleation sites and diffusion pathways for Na^+^. The combination of the copper nanowires and porous copper foam enabled uniform charge distribution and confined sodium deposition within the conducting copper nanowires. As a result, the SMB exhibited a prolonged cycle life of over 1,000 h and a high Coulomb efficiency of more than 97.5% (**Figure** **7**a).^[^ ^69^ ^]^

Zhou utilized a 3D nickel foam skeleton modified with nitrogen‐doped graphene for sodium plating. The nitrogen‐containing functional groups in the graphene structure acted as nitriphilic sites, reducing the nucleation overpotential of sodium and guiding the uniform flux of Na^+^. This modification led to a regularized electric field distribution, effectively inhibiting the formation of sodium dendrites (Figure 7c).^[^ ^70^ ^]^

While the 3D metal matrix modification methods mentioned above have demonstrated improved dendrite‐free deposition and excellent electrochemical performance, the weight and cost of the metal materials can be relatively high. In terms of battery design, the increase in inert material weight can hinder the achievement of high energy density. Consequently, researchers have increasingly focused on carbon skeleton matrices as promising alternatives for SMBs.

#### 3.3.2 Carbon 3D Skeleton

Carbon materials are widely considered excellent framework materials for alkali metal deposition, including sodium, due to their numerous benefits. These advantages encompass their lightweight characteristics, high electrical conductivity, exceptional chemical stability, and cost‐effectiveness in manufacturing.

Luo inhales melted sodium into the Spaces between sheets of reduced graphene oxide (RGO) to prepare a composite metal sodium negative electrode, which can be molded into a variety of shapes, such as a one‐dimensional monomer of controllable size, a two‐dimensional film, or a 3D composite negative electrode (Figure 7b).^[^ ^71^ ^]^

Hu et al. encapsulated sodium metal into a conductive body of carbonized natural wood with porous channels to obtain a composite electrode of sodium carbonized wood (Na‐wood). After long‐term cycling, sodium metal remained stable in the channels of carbonized wood. Thanks to this unique structure, the sodium/carbonized wood composite electrode has a potential of 30 mV at 0.5 mA cm^−2^ current density and a cycle life of over 500 h at 1.0 mA cm^−2^ (Figure 7d).^[^ ^72^ ^]^

The above methods achieve the dendrite‐free Na deposition through the homogenized distribution of molten Na on the carbon skeleton composite, to a large extent stabilizing the configuration of metallic anode, and alleviating the dramatic volume change upon the repetitive Na deposition and stripping process. However, the 3D composite negative electrode may face the problem of structural failure during the long‐term cycle, which is mainly due to the local stress concentration caused by the uneven deposition of sodium metal. Another method is to uniformly deposit sodium metal on the surface of the 3D collector fluid by electrodeposition. However, due to the lattice mismatch between s*p* ^2^/s*p* ^3^ hexagonal carbon and body‐centered cubic (bcc) metallic sodium, the carbon material is sodiophilic, which is especially fatal for electrodeposition.

## 4 Outlook and Future Prospects

In this review, multiscale effective strategies have been reviewed to promote the Na^+^ utilization efficiency. Despite performance progress achieved, future research endeavors should focus on more systematic strategies and precise mechanism analysis described as follows: 1) Cation utilization in SMBs is not solely determined by individual components but rather relies on the interplay between the cathode, anode, electrolyte/quasi‐solid electrolyte, and separator. Therefore, the reliable control of the electrolyte/ electrode/ interface alone cannot reflect the overall performance on the cell level. In this regard, the comprehensive analysis along the Na^+^ migration pathway is required, especially in consideration of specific cell models and dimensional specifications; 2) In addition to electrode/electrolyte/interface modification, the separator is also crucial for facilitating efficient Na^+^ transfer. However, most laboratory evaluations still employ traditional glass fiber or polyethylene (PE) in the SMB prototypes. The hydrophobic nature, low porosity, poor wettability, and thermal stability of these separators would increase the cell resistance, exacerbate sodium dendrite formation, and contribute to internal short circuits and thermal runaway. Surface modifications of the polyolefin membranes could provide a general strategy to optimize the physicochemical properties of the separators such as ionic conductivity, sodium transference number, porosity, and electrolyte wettability. If the interfacial behavior of electrodes can be regulated by constructing a multifunctional coating on the separator as in the contact with the electrodes, then the Na^+^ utilization optimization could be achieved through the facile separator modification in a lower cost; 3) Operating conditions have a significant impact on the cation utilization degree. Parameters such as temperature and pressure influence cycle performance, but their effects are often intertwined during the operation. Mechanism validation requires cell sample holders that operate under quantitatively varied physical fields, such as a wide temperature range, pressure gradient, or high/low current density, to decouple these parameters. For instance, the parameter of temperature alone would affect cation diffusion in the bulk solution, desolation processes at the interface, and solid‐state diffusion; meanwhile, a series of thermodynamics (Gibbs formation energy of the alloy nucleation sites, sodiophilicity) and kinetics factors (interfacial diffusivity, current density) would also be involved in each process. Defining the individual and combined effects of multiple physical factors on a continuous process requires the step‐by‐step decoupling of complex conditions; and 4) Due to the complex interactions between electrodes and electrolytes, simulations of the practical conditions of sodium dendrite growth are rather challenging. Therefore, the development of in situ/operational methods to observe the sodium metal nucleation and propagation process, and real‐time interfacial and phasic changes of the as‐paired cathode are necessary. Considerations should include avoiding interface changes or damage during the sample processing, quantifying the threshold of X‐ray and electron flux dose to avoid the interaction with sodium metal to prevent structural damage, as well as examining key properties of dendrite nucleation and expansion at multiple scales, for instance, the atomic‐level interface reactions, solid‐state ion transport in the bulk electrode level, and interactions with other components (electrolyte and cathode) within the device.

## 5 Conclusion

This review article has provided a comprehensive analysis of strategies to promote cation utilization in energy‐dense SMB prototypes. By identifying various influencing factors associated with the sodium metallic deposition process, particularly the root origins of nucleation and deposits propagation and their correlations, we have highlighted key features on the electrode level that contribute to the enhanced cation utilization. Additionally, the impact of working conditions and parameters, such as temperature, current density, and areal capacity, on battery performance has been emphasized. These factors play a crucial role in achieving optimal cation utilization and overall battery efficiency.

Furthermore, the review has summarized the promising strategies to improve cation utilization. These include the development of advanced electrolyte formulations, the design of novel electrode materials, and the exploration of innovative battery architectures. These strategies offer potential solutions to mitigate dendrite formation and enhance overall battery performance. Looking forward, there are still many areas that deserve further research and exploration. Understanding the stepwise nucleation and dendrite growth models with real‐time characterizations as well as optimizing the high‐rate ion pathway across the multiple electrode/electrolyte interfaces are areas of great importance. Additionally, the scalability, cost‐effectiveness, and long‐term stability of energy‐dense SMB prototypes should be thoroughly investigated to ensure their practical viability.

In summary, this review has provided valuable insights into promoting cation utilization in energy‐dense SMB prototypes. By addressing the challenges and exploring the complementary mitigation strategies, we are optimistic about the prospects of SMB systems. Continued research and development efforts in this field will contribute to the realization of efficient, sustainable, and high‐performance energy storage technologies for a greener future.

## Conflict of Interest

The authors declare no conflict of interest.
